# Glyceraldehyde 3-Phosphate Dehydrogenase and Galectin from *Dirofilaria immitis* Excretory/Secretory Antigens Activate Proangiogenic Pathway in In Vitro Vascular Endothelial Cell Model

**DOI:** 10.3390/ani15070964

**Published:** 2025-03-27

**Authors:** Manuel Collado-Cuadrado, Alfonso Balmori-de la Puente, Iván Rodríguez-Escolar, Elena Infante González-Mohino, Claudia Alarcón-Torrecillas, Miguel Pericacho, Rodrigo Morchón

**Affiliations:** 1 Zoonotic Diseases and One Health Group, Faculty of Pharmacy, Biomedical Research Institute of Salamanca (IBSAL), Centre for Environmental Studies and Rural Dynamization (CEADIR), University of Salamanca, 37007 Salamanca, Spain; manuelcollado@usal.es (M.C.-C.); a.balmori@usal.es (A.B.-d.l.P.); ivanrodriguez@usal.es (I.R.-E.); elena.igm4@usal.es (E.I.G.-M.); 2 Department of Physiology and Pharmacology, Biomedical Research Institute of Salamanca (IBSAL), University of Salamanca, 37007 Salamanca, Spain; claudia3alarcon@usal.es (C.A.-T.); pericacho@usal.es (M.P.)

**Keywords:** angiogenesis, *Dirofilaria immitis* excretory/secretory antigens, cellular processes, galectin, glyceraldehyde 3-phosphate dehydrogenase

## Abstract

Heartworm disease is caused by *Dirofilaria immitis*, which mainly affects canids and felids. Adult *D. immitis* worms are located between the heart’s right ventricle and the pulmonary artery. These parasites produce an inflammatory and hypoxic process in the vascular endothelium. It has been demonstrated that *D. immitis* excretory/secretory antigens are able to stimulate the angiogenic process as a survival mechanism of *D. immitis* in the vascular endothelium, stimulating the proangiogenic pathway and related cellular processes. Our goal was to study the role of glyceraldehyde 3-phosphate dehydrogenase (GAPDH) and galectin (GAL) (proteins of *D. immitis* excretory/secretory antigens) plus vascular endothelial growth factor isoform A (VEGF-A) in the angiogenic process and their relationship with three cellular processes (cell proliferation, cell migration, and pseudocapillary formation) in an in vitro model of vascular endothelial cells. Cell viability and cytotoxicity were analyzed by live cell analysis and a commercial kit, respectively. VEGF-A, sVEGFR-2, VEGFR-1/sFlt, soluble endoglin, and membrane endoglin were analyzed by commercial ELISA kits. Cell proliferation, cell migration, and pseudocapillary formation were analyzed by MTT-based assay, the wound healing technique, and counting cell connections and cell clusters, respectively. rDiGAPDH+VEGF-A and rDiGAL+VEGF-A significantly increased the expression of sVEGFR-2, mEndoglin, and VEGF-A compared to cultures treated with only the proteins (rDiGAPDH and rDiGAL), VEGF-A, or unstimulated cultures. In addition, they also produced a significant increase in cell proliferation, cell migration, and pseudocapillary formation. Therefore, these proteins together with VEGF-A can activate the proangiogenic pathway and could be related to *D. immitis* survival in the circulatory system.

## Simple Summary

*Dirofilaria immitis* is the causative agent of heartworm disease and mainly affects domestic dogs, among others, causing an acute inflammatory pathology. In this study, the effects of two *D. immitis* proteins, which are secreted into the bloodstream, on the vascular endothelium and parasite survival were analyzed. Our results highlight the significant increase in the production of molecules and cellular processes related to the formation of *D. immitis*.

## 1. Introduction

Angiogenesis is a complex and dynamic process in which new blood vessels form from existing ones. It naturally occurs during embryonic development and can also be triggered in pathological conditions by factors like hypoxia, inflammation, or tissue damage [1]. Its activation triggers a series of morphogenetic changes affecting endothelial cells [2,3]. The vascular endothelial growth factor (VEGF) is one of the most important molecules that regulates this process. It comprises a group of isoforms, VEGF-A being the main one [4]. Depending on which receptors it binds to (VEGFR-1, VEGFR-2, or VEGFR-3), it emits signals of activation or restriction of the angiogenic process [5]. In response to stimuli such as inflammation and hypoxia, there is an increase in VEGF-A expression and endothelial cells are transformed into filopodia structures with migratory capacity whose direction is determined by the VEGF-A gradient [6,7,8]. When VEGF-A binds to VEGFR-2, endothelial cell proliferation, migration, and vascular permeability are enhanced [4,9], resulting in a proangiogenic response [10]. On the other hand, when it binds to the VEGFR-1 receptor and sFlt1 (soluble form), it produces a negative regulation by preventing its binding to the VEGFR-2 receptor, decreasing proangiogenic signals [11]. Another key molecule in angiogenesis is endoglin (TGF-β co-receptor), which promotes endothelial cell proliferation via membrane endoglin (mEndoglin). However, when soluble endoglin (sEndoglin) appears through the action of metalloproteinases, it stimulates vasoconstriction and facilitates the antiangiogenic pathway [12,13,14,15,16].

*Dirofilaria immitis* causes heartworm disease (a vector-borne zoonotic disease). This nematode parasite affects domestic dogs and cats and other wild canids and felids (foxes, coyotes, wolves, lynxes, etc.) [17]. It is a chronic, severe, and potentially fatal disease with a progressive and complex course that, in its late stages, can cause the death of the infected host [18,19]. Damage to the vascular endothelium and lung tissue is mainly caused by the presence of pre-adult and adult worms in the arterio-pulmonary network, right ventricle of the heart, and adjacent large vessels. Alongside chronic progression, acute events may arise due to the natural death of adult *D. immitis* worms or as a result of filarial treatment. This leads to the release of antigenic components from the parasite and the endosymbiotic bacteria *Wolbachia* spp. into the bloodstream, triggering intensified inflammatory responses in the vascular endothelium and the development of significant thromboemboli. Together, these will accelerate congestive heart failure and the animal’s death [20,21,22,23]. On the other hand, microfilariae of *D. immitis* and *Wolbachia* spp. can cause severe renal dysfunction leading to membranous glomerulonephritis and proteinuria [24,25,26].

On the one hand, the presence of *D. immitis* causes the enlargement of endothelial cells and intercellular spaces in the vascular endothelium, which becomes disorganized. Additionally, the medial elastic lamina tends to degenerate, increasing vascular permeability and altering the elasticity of the walls. This favors smooth muscle cell migration and induces intravascular microvilli formation (proliferative endoarteritis). All these changes lead to decreased blood flow, hypoxia, chronic pulmonary oedema, luminal obstruction, hypertension, and congestive heart failure [19,27,28,29,30,31]. On the other hand, *D. immitis* can survive for years within the host, successfully evading both inflammatory and immune responses. To this end, it has developed different processes that contribute to its survival and minimize disease-associated pathology through the action of metabolic products excreted and/or secreted into the bloodstream on the vascular endothelium [19,32,33]. These processes include the activation of nitric-oxide-dependent endothelial relaxation mechanisms [27,28], stimulation of the synthesis of cyclooxygenase-2 (COX-2), and consequent production of prostaglandin E_2_ (PGE_2_), with vasodilator activity, decreased monocyte transmigration in vascular endothelial cells [34], and interaction with the fibrinolytic system increasing plasmin levels and limiting clot formation [35,36]. For instance, the galectin (GAL) and glyceraldehyde-3-phosphate dehydrogenase (GAPDH) proteins of the *D. immitis* excretory/secretory antigens are able to activate, through the production of plasmin, the proliferation and migration of endothelial and muscle cells while being able to initiate the degradation of the extracellular matrix, which, in the long term, could favor the appearance of proliferative endoarteritis [37,38].

It has recently been shown that *D. immitis* excretory/secretory antigens and *Dirofilaria repens* somatic antigens can stimulate the angiogenic pathway, the expression of proangiogenic molecules, and a number of cellular processes related to this pathway as a possible survival mechanism [39,40]. Considering this fact and that GAL and GAPDH proteins are part of the *D. immitis* excretory/secretory antigens, our goal was to study the role of these proteins in the angiogenic pathway through the analysis of pro- and antiangiogenic angiogenic molecules and some cellular processes directly related to this process.

## 2. Materials and Methods

### 2.1. Cell Culture

A primary culture of human umbilical vein endothelial cells (HUVECs) was cultured as described by Machado et al. [39]. HUVECs were maintained at 37 °C with 5% CO_2_ and 95% air in Endothelial Basal Medium 2 supplemented with SingleQuots^®^ (Lonza, Walkersville, MD, USA): 20% fetal bovine serum, 22.5 µg/mL, heparin (22.5 µg/mL), VEGF (0.5 ng/mL), ascorbic acid (1 µg/mL), hFGF-B (10 ng/mL), hydrocortisone (0.2 µg/mL), hEGF (5 ng/mL), gentamicin (30 mg/mL), amphotericin B (15 µg/mL), and R^3^-IGF-3 (20 ng/mL). Cells were grown on plates pre-coated with pig gelatin (0.1%), fibronectin (0.01%) (Sigma-Aldrich, St. Louis, MA, USA), and collagen (0.01%) (Corning, New York, NY, USA), with the medium changed every three days. Expansion was performed by trypsinization at a 1:3 ratio.

### 2.2. Reagents

*D. immitis* excretory/secretory antigens (DiES) were prepared following González-Miguel et al. [35]. Briefly, 23 live adult *D. immitis* worms (8 ♂/15 ♀) were washed and incubated in Eagle’s Minimum Essential Medium with gentamicin (0.04%) and nystatin (0.01%). The collected medium was processed in the following manner in the same way [35], resulting in a final concentration of 1.3 µg/µL. Adult *D. immitis* worms were extracted for the purpose of routine veterinary work.

GAPDH (rDiGAPDH) and GAL (rDiGAL) proteins belonging to DiES were recombinantly produced following the methodology described [38]. In summary, first-strand cDNA and the cDNA sequences of GAPDH and GAL were amplified using the following primers: GAPDHRev (5′-TTATCTGCTGGCGATGTAAGAGAG), GAPDHFwd (5′-CTAGTGCATTTGAATACCGCTCACTTC). GALFwd (5′-ATGCACCACACAACGAATGAAACGAATTAC), and GALRev (5′-ATGAGCAAACCAAACCAAAGATTGGAATC). PCR amplifications were performed in 1 cycle at 94 °C for 5 m, 35 cycles at 94 °C for 1 m, 46 °C for 46 s, and 72 °C for 1 min 30 s and 1 cycle at 72 °C for 5 m. PCR products were loaded on agarose gel and electrophoresis was performed and the bands were purified using the StrataPrep DNA Gel Extraction Kit (Stratagene, San Diego, CA, USA). DiGAPDH and DiGAL products were then cloned into the pSC-A vector using the StrataClone PCR Cloning kit (Stratagene, USA) and purified using the Machery-Nagel NuceoSplin Plasmid kit (Machery-Nagel, Düren, Germany). For expression and purification of DiGAPDH and DiGAL, PCR products containing the coding sequence of the proteins were cloned into the TOPO vector (Invitrogen, Waltham, MA, USA) following the manufacturer’s instructions. Recombinant plasmids were transformed into *Escherichia coli* XL1B strains grown on LB-agar plates with amplicin (100 μg/mL). Vectors containing the sequence of interest in the correct reading frame were transformed into BL-21 cells in liquid LB plus amplicin (100 μg/mL), and subsequently, protein expression was induced by 0.2% L-arabinose. Transfected cells were sonicated in buffer containing 8M urea, 100 mM NaH_2_PO_4_, and 10 mM Tris-Cl, pH 7.9. After a 20 min centrifugation step at 10,000× *g*, the supernatant was applied to the HIS-Select Nickel affinity gel (Sigma-Aldrich, MA, USA) for protein purification, following the manufacturer’s instructions. Subsequently, rDiGAPDH and rDiGAL were eluted in elution buffer (50 mM NaH_2_PO_4_, 300 mM NaCl, and 250 mM imidazole, pH 7.9) and dialysed in PBS and then stored until use at −80 °C. Finally, rDiGAPDH and rDiGAL were tested for the presence of endotoxin contamination using a quantitative *Limulus* Amebocyte Lysate test QCL 1000 (BioWhittaker, Walkersville, MD, USA). The endotoxin quantity was under the sensitivity level of cell stimulation (<0.4 U/mg protein).

### 2.3. Endothelial Cell Stimulation, Citotoxity and Cell Viability

HUVECs were treated as previously described [39]. In brief, endothelial cells were cultured in 60 mm plates and allowed to grow to confluent cultures and then treated for 24 h with VEGF-A (R&D Systems, Minneapoli, MN, USA), rDiGAPDH, rDiGAL, rDiGAPDH+VEGF-A, and rDiGAL+VEGF-A at 1 μg/mL for each of the molecules used. Unstimulated cell cultures were used as negative control and cell cultures stimulated with DiES+VEGF-A (1 μg/mL each) were used as positive control (Control+) [38]. Finally, the supernatant was collected, and cells were lysed using lysis buffer (20 mM Tris-HCl pH 7.5, 140 mM NaCl; 10 mM ethylenediaminetetraacetic acid, 10% glycerol, 1% Igepal CA-630, aprotinin, pepstatin, and leupeptin at 1 μg/mL each; 1 mM phenylmethlsulfonyl fluoride and 1 mM sodium orthovanadate).

Cell viability was measured by counting cells using the Countess Automated Cell Counter (Invitrogen, USA) according to the manufacturer’s instructions. Cytotoxicity was analyzed from the supernatant of stimulated and control cultures using the Toxilight BioAssay Kit (Cambrex, Liège, Belgium) according to the manufacturer’s instructions. This commercial kit quantitatively measures adenylate cyclase release from damaged cells. Results are presented as the means ± standard deviations (SDs) of three experiments performed in duplicate.

### 2.4. Angiogenic Factors Assays

The concentrations of VEGF-A, VEGFR-1/sFlt, sVEGFR-2, and sEndoglin in the endothelial cell culture medium and mEndoglin in the endothelial cells lysed were measured using the Human Quantikine ELISA kit (R&D Systems, USA) following the manufacturers’ instructions. The results are presented as the means ± SDs of three experiments performed in duplicates.

### 2.5. Proliferation, Migration, and Endothelial Cell Tube Formation Assays

Proliferation, migration, and endothelial cell tube formation assays were conducted with some modifications based on previous methods [40]. Cultures without treatment served as the negative control while cultures treated with DiES+VEGF-A acted as the positive control (Control+) [39], under identical conditions.

Proliferation assay was assessed every 2 days for 10 days by incubating the cultures with 0.5 mg/mL MTT (Sigma-Aldrich, USA) for 4 h. After incubation, 10% SDS in 0.01 M HCl (1:1, *v*/*v*) was added (overnight, 37 °C) and the absorbance was measured (595 nm). For the migration assay (wound healing), the same methodology was followed by monitoring every 30 min under the inverted phase contrast microscope. Finally, for pseudocapillary formation assays, Ibidi Angiogenesis Slide plates (15-well, 0.23 cm^2^) pre-coated with Corning^®^ Matrigel^®^ Basement Membrane Matrix (Merck, Darmstadt, Germany) were used. Cells (7.5–9 *×* 10^3^ cells/well) were seeded and treated with the different conditions. Cell bodies and connections were analyzed 90 min post treatment by imaging with an inverted microscope. The ratio of intercellular connections to cell bodies was calculated.

All results are expressed as the means ± SDs of three independent experiments.

### 2.6. Data Analysis

Analyses were performed with R software (R Core Team 2023) by ANOVA, checking the following assumptions: |skeweness and kurtosis| < 2 and Shapiro test (*p* > 0.05); homoscedasticity with Levene test (*p* > 0.05); and normally distributed residuals with Shapiro test (*p* > 0.05).

Whenever significant differences were detected, post hoc comparisons of individual means were conducted using Tukey’s test. Data are presented as means ± SDs. In all experiments, statistical significance was considered at *p* < 0.05 (95% confidence level) and <0.01 (99% confidence level).

## 3. Results

### 3.1. Effect of rDiGAPDH and rDiGAL on Cell Viability, Cytotoxicity, and Angiogenic Factors via VEGF-A

**Figure 1:**
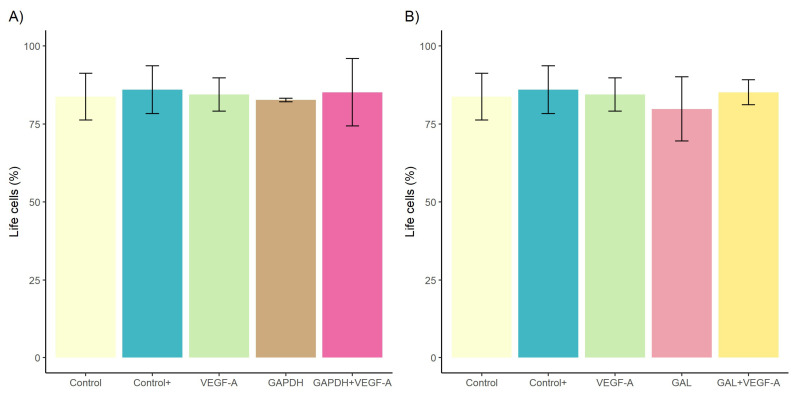
Effects of rDiGAPDH (**A**) and rDiGAL (**B**) on cell viability and cytotoxicity in control (light yellow), Control+ (DiES+VEGF-A) (blue), and cultures stimulated with VEGF-A (green), rDiGAPDH (brown), rDiGAPDH+VEGF-A (dark pink), rDiGAL (light pink) and rDiGAL+VEGF-A (dark yellow) during the first 24 h. The results are presented as the means ± SDs of 3 independent experiments.

No differences in cell viability and cytotoxicity were observed among cell cultures stimulated with VEGF-A, rDiGAPDH, rDiGAPDH+VEGF-A, and DiES+VEGF-A (positive control) and non-stimulated cultures (control) (*F* = 0.093, *df* = 4, *p* > 0.05 in all cases) and between cell cultures stimulated with VEGF-A, rDiGAL, rDiGAL+VEGF-A, and the positive control and control (*F* = 0.324, *df* = 4, *p* > 0.05 in all cases) (Figure 1).

**Figure 2:**
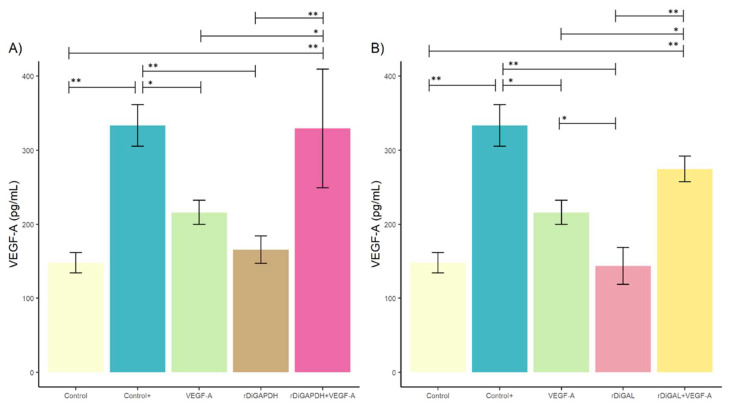
Effects of rDiGAPDH (**A**) and rDiGAL (**B**) on VEGF-A production in control (light yellow), Control+ (DiES+VEGF-A) (blue), VEGF-A (green), rDiGAPDH (brown), rDiGAPDH+VEGF-A (dark pink), rDiGAL (light pink), and rDiGAL+VEGF-A (dark yellow) during the first 24 h. The results are presented as the means ± SDs of 3 independent experiments. Asterisks indicate significant differences: *p* < 0.05 (*); *p* < 0.01 (**).

Regarding VEGF-A production (Figure 2), the stimulation of cell cultures with rDiGAPDH+VEGF-A produced significant differences between cultures stimulated with rDiGAPDH (*F* = 14.66, *df* = 4, *p* = 0.0037), VEGF-A (*F* = 14.66, *df* = 4, *p* = 0.0380), and control (*F* = 14.66, *df* = 4, *p* = 0.0017). Similarly, non-significant values were observed in the positive control (*F* = 14.66, *df* = 4, *p* = 0.9999) (Figure 2A).

Cell cultures stimulated with rDiGAL+VEGF-A also exhibited in a significant increase in VEGF-A production compared to cultures stimulated with rDiGAL (*F* = 46.35, *df* = 4, *p* = 0.0001) and VEGF-A (*F* = 46.35, *df* = 4, *p* = 0.0397) and non-stimulated cultures (*F* = 46.35, *df* = 4, *p* = 0.0001) (Figure 2B). However, no differences were reported with DiES+VEGF-A treatment (positive control) (*F* = 46.35, *df* = 4, *p* = 0.3709). VEGF-A-stimulated cell cultures showed a significant increase in VEGF-A production compared to rDiGAL (*F* = 46.35, *df* = 4, *p* = 0.0152) and not in the rest of the treated and untreated cell cultures (*F* = 46.35, *df* = 4, *p* > 0.05). In addition, the positive control showed significant differences with cultures treated with VEGF-A (*F* = 14.66, *df* = 4, *p* = 0.0312), rDiGAPDH (*F* = 14.66, *df* = 4, *p* = 0.0031), and rDiGAL (*F* = 46.35, *df* = 4, *p* < 0.0001) and non-stimulated cultures (*F* = 14.66, *df* = 4, *p* = 0.0014).

**Figure 3:**
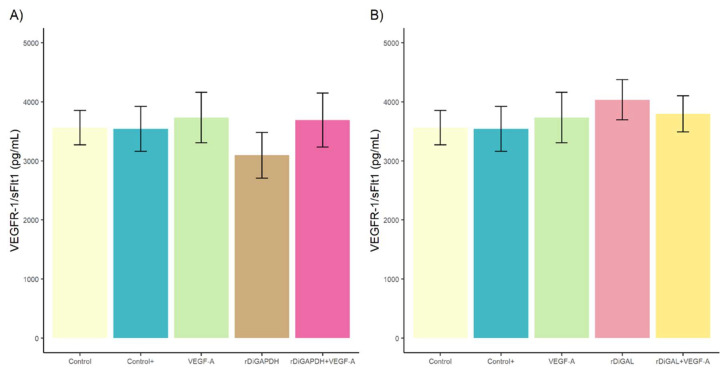
Effects of rDiGAPDH (**A**) and rDiGAL (**B**) on VEGFR-1/sFlt1 production in control (light yellow), Control+ (DiES+VEGF-A) (blue), VEGF-A (green), rDiGAPDH (dark), rDiGAPDH+VEGF-A (dark pink), rDiGAL (light pink), and rDiGAL+VEGF-A (dark yellow) during the first 24 h. The results are presented as the means ± SDs of 3 independent experiments.

With respect to VEGFR-1/sFlt concentration, no significant differences were observed between stimulated and unstimulated cultures (Figure 3) (for rDiGAPDH, *F* = 1.254, *df* = 4, and *p* > 0.05 in all cases, and for rDiGAL, *F* = 0.968, *df* = 4, and *p* > 0.05 in all cases).

**Figure 4:**
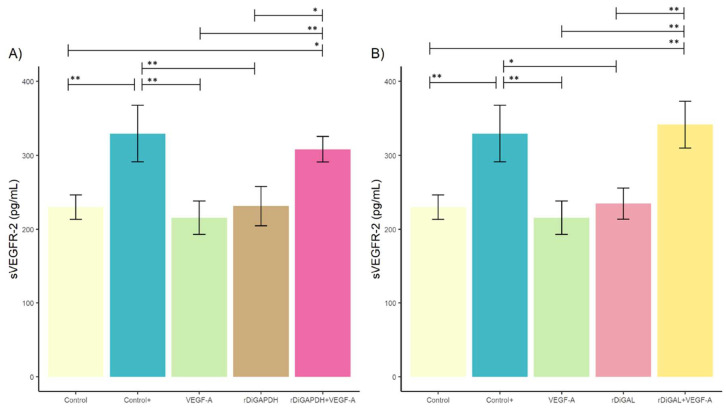
Effects of rDiGAPDH (**A**) and rDiGAL (**B**) on sVEGFR-2 production in control (light yellow), Control+ (DiES+VEGF-A) (blue), VEGF-A (green), rDiGAPDH (brown), rDiGAPDH+VEGF-A (dark pink), rDiGAL (light pink), and rDiGAL+VEGF-A (dark yellow) during the first 24 h. The results are presented as the means ± SDs of 3 independent experiments. Asterisks indicate significant differences: *p* < 0.05 (*); *p* < 0.01 (**).

Nevertheless, sVEGFR-2 concentration was significantly higher in cultures stimulated with rDiGAL+VEGF-A and rDiGAPDH+VEGF-A compared to those stimulated with rDiGAL (*F* = 14.69, *df* = 4, *p* = 0.0049) and rDiGAPDH (*F* = 12.47, *df* = 4, *p* = 0.0267), respectively (Figure 4). A similar event happened when comparing cultures stimulated with VEGF-A (*F* = 14.69, *df* = 4, *p* = 0.0014 and *F* = 12,47, *df* = 4, *p* = 0.0085) and the control (*F* = 14.69, *df* = 4, *p* = 0.0036 and *F* = 12.47, *df* = 4, *p* = 0.0241) (Figure 4). There were no significant differences between Control+ and cultures stimulated with rDiGAPDH+VEGF-A (*F* = 12.47, *df* = 4, *p* = 0.8387) and rDiGAL+VEGF-A (*F* = 14.69, *df* = 4, *p* = 0.9804). On the other hand, sVEGFR-2 concentration was significantly higher in Control+ compared with cultures stimulated with VEGF-A (*F* = 12.47, *df* = 4, *p* = 0.0085), rDiGAPDH (*F* = 12.47, *df* = 4, *p* = 0.0057), and rDiGAL (*F* = 14.69, *df* = 4, *p* = 0.0109) and the control (*F* = 12.47, *df* = 4, *p* = 0.0051). There were no significant differences between control and cultures treated with VEGF-A and rDiGAPDH (*F* = 12.47, *df* = 4, *p* > 0.05 in all cases). No differences were reported between the control and cultures treated (VEGF-A and rDiGAL) (*F* = 14.69, *df* = 4, *p* > 0.05 in all cases).

### 3.2. Effect of rDiGAPDH and rDiGAL on Endoglin Production

**Figure 5:**
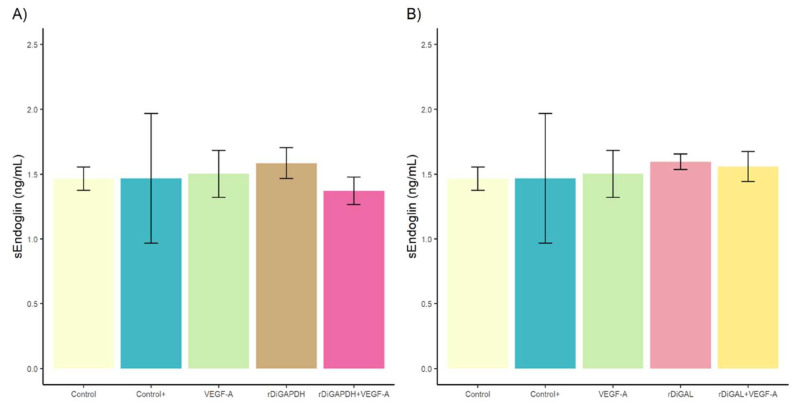
Effects of rDiGAPDH (**A**) and rDiGAL (**B**) on sEndoglin production in control (light yellow), Control+ (DiES+VEGF-A) (blue), VEGF-A (green), rDiGAPDH (brown), rDiGAPDH+VEGF-A (dark pink), rDiGAL (light pink), and rDiGAL+VEGF-A (dark yellow) during the first 24 h. The results are presented as the means ± SDs of 3 independent experiments.

No significant differences were detected in sEndoglin production between stimulated and non-stimulated cultures (for rDiGAPDH, *F* = 0.282, *df* = 4, and *p* > 0.05 in all cases, and for rDiGAL, *F* = 0.163, *df* = 4, and *p* > 0.05 in all cases) (Figure 5) (*F* = 15.87, *df* = 4, *p* = 0.002).

**Figure 6:**
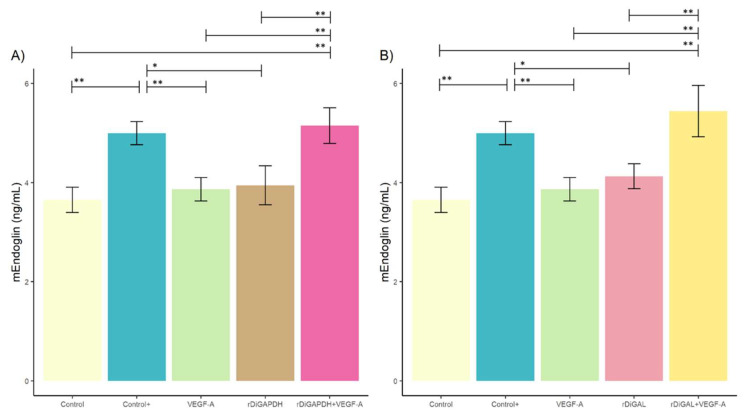
Effects of rDiGAPDH (**A**) and rDiGAL (**B**) on mEndoglin production in control (light yellow), Control+ (DiES+VEGF-A) (blue), VEGF-A (green), rDiGAPDH (brown), rDiGAPDH+VEGF-A (dark pink), rDiGAL (light pink), and rDiGAL+VEGF-A (dark yellow) during the first 24 h. The results are presented as the means ± SDs of 3 independent experiments. Asterisks indicate significant differences: *p* < 0.05 (*); *p* < 0.01 (**).

In contrast, significant differences in mEndoglin production were observed in cultures stimulated with rDiGAPDH+VEGF-A compared to those treated with VEGF-A (*F* = 15.87, *df* = 4, *p* = 0.0028), and rDiGAPDH (*F* = 15.87, *df* = 4, *p* = 0.0004) and the control (*F* = 15.87, *df* = 4, *p* = 0.0008) (Figure 6A). Similarly, cultures stimulated with rDiGAL+VEGF-A exhibited significant differences when compared to those treated with rDiGAL (*F* = 17.53, *df* = 4, *p* = 0.0034) and VEGF-A (*F* = 17.53, *df* = 4, *p* = 0.0008) and the control (*F* = 17.53, *df* = 4, *p* = 0.0003) (Figure 6B). However, no significant differences were found between cultures treated with the positive control and those stimulated with rDiGAPDH+VEGF-A (*F* = 15.87, *df* = 4, *p* = 0.9675) or rDiGAL+VEGF-A (*F* = 17.53, *df* = 4, *p* = 0.4646). Additionally, no significant differences were noted between the control and those cultures treated with VEGF-A (*F* = 15.87, *df* = 4, *p* = 0.9066), rDiGAPDH (*F* = 15.87, *df* = 4, *p* = 0.7598), or rDiGAL (*F* = 17.53, *df* = 4, *p* = 0.4125). In contrast, significant differences were found between the positive control and cultures stimulated with VEGF-A (*F* = 15.87, *df* = 4, *p* = 0.0069), rDiGAPDH (*F* = 15.87, *df* = 4, *p* = 0.0114), and rDiGAL (*F* = 17.53, *df* = 4, *p* = 0.0045) and the control.

### 3.3. Effect of Antigens on Cell Proliferation and Migration

**Figure 7:**
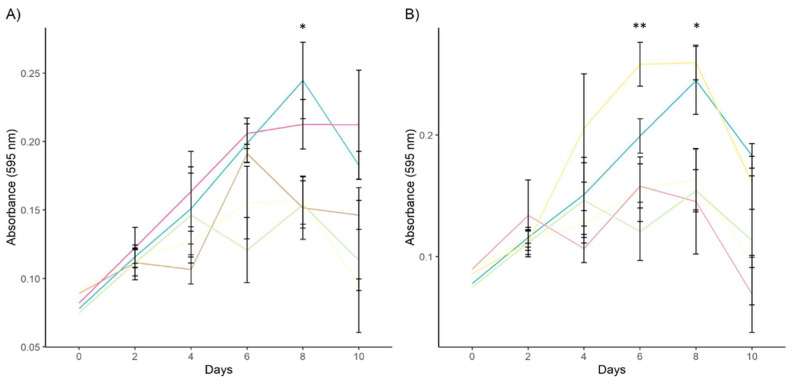
Effects of rDiGAPDH (**A**) and rDiGAL (**B**) on cell proliferation at 10 days in control (light yellow), Control+ (DiES+VEGF-A) (blue), VEGF-A (green), rDiGAPDH (brown), rDiGAPDH+VEGF-A (dark pink), rDiGAL (light pink), and rDiGAL+VEGF-A (dark yellow). The results are presented as the means ± SDs of 3 independent experiments. Asterisks indicate significant differences: *p* < 0.05 (*); *p* < 0.01 (**).

Both stimulated and unstimulated cell cultures exhibited a typical growth curve, with an increase in cell numbers from days 0 and 8 post stimulation followed by a decline from day 10 onward (Figure 7). Treatment with rDiGAPDH+VEGF-A significantly improved cell viability compared to cultures stimulated with rDiGAPDH alone (*F* = 12.02, *df* = 4, *p* = 0.0345), VEGF-A (*F* = 12.02, *df* = 4, *p* = 0.0425) and the control (*F* = 12.02, *df* = 4, *p* = 0.05) at 8 days post stimulation. However, no significant differences were detected between cultures stimulated with rDiGAPDH+VEGF-A and the positive control (*F* = 12.02, *df* = 4, *p* = 0.3986) or between cultures treated with VEGF-A and rDiGAPDH and the control (*F* = 12.02, *df* = 4, *p* > 0.05 in all cases).

When cultures were treated with rDiGAL+VEGF-A, a significant increase in viable cell numbers was observed at 6 days post stimulation compared to those treated with rDiGAL (*F* = 19.51, *df* = 4, *p* = 0.001), VEGF-A (*F* = 19.51, *df* = 4, *p* < 0.0001) and the control (*F* = 19.51, *df* = 4, *p* = 0.0008). Similarly, at 8 days post stimulation, the number of viable cells remained significantly higher compared to those in cultures treated with rDiGAL (*F* = 11.66, *df* = 4, *p* = 0.0033) and VEGF-A (*F* = 11.66, *df* = 4, *p* = 0.0058) and the control (*F* = 11.66, *df* = 4, *p* = 0.0111). No significant differences were observed between cultures stimulated with rDiGAL+VEGF-A and the positive control (*F* = 11.66, *df* = 4, *p* > 0.05). Additionally, no significant differences were found between cultures stimulated with VEGF-A and rDiGAL and the control (*F* = 11.66, *df* = 4, *p* > 0.05 in all cases) at 6-8 days after the stimulation.

**Figure 8:**
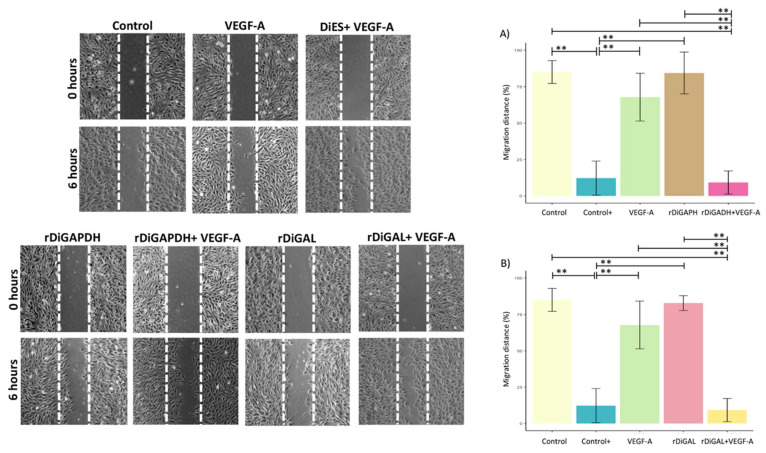
Representative images and effects of rDiGAPDH (**A**) and rDiGAL (**B**) on cell migration distance in control (light yellow), Control+ (DiES+VEGF-A) (blue), VEGF-A (green), rDiGAPDH (brown), rDiGAPDH+VEGF-A (dark pink), rDiGAL (light pink), and rDiGAL+VEGF-A (dark yellow) during the first 6 h. The results are presented as the means ± SDs of 3 independent experiments. Asterisks indicate significant differences: *p* < 0.01 (**).

With respect to cell migration, a significant reduction in migration distance (Figure 8) was observed in cultures stimulated with rDiGAPDH+VEGF-A and rDiGAL+VEGF-A compared to those treated with rDiGAPDH (*F* = 49.43, *df* = 4, *p* < 0.0001) and rDiGAL (*F* = 64.23, *df* = 4, *p* < 0.0001), respectively. Conversely, a significant increase in migration distance was noted in comparison to cultures stimulated with VEGF-A (*F* = 49.43, *df* = 4, *p* < 0.0001 and *F* = 64.23, *df* = 4, *p* < 0.0001) and the control (*F* = 49.43, *df* = 4, *p* < 0.0001 and *F* = 64.23, *df* = 4, *p* < 0.0001). No significant differences were detected between cultures treated with rDiGAPDH+VEGF-A and those receiving the positive control (*F* = 49.43, *df* = 4, *p* = 0.9946). Similarly, no significant differences were found between the control and those treated with VEGF-A (*F* = 49.43, *df* = 4, *p* = 0.2042) or rDiGAPDH (*F* = 49.43, *df* = 4, *p* = 0.9999). Likewise, no significant variations were observed between cultures stimulated with rDiGAL+VEGF-A and the positive control (*F* = 64.23, *df* = 4, *p* = 0.9908), nor between the control and those treated with VEGF-A (*F* = 64.23, *df* = 4, *p* = 0.2018) or rDiGAL (*F* = 64.23, *df* = 4, *p* = 0.9972). However, significant differences were found between the positive control and cultures stimulated with VEGF-A (*F* = 49.43, *df* = 4, *p* < 0.0001), rDiGAPDH (*F* = 49.43, *df* = 4, *p* < 0.0001), and rDiGAL (*F* = 64.23, *df* = 4, *p* < 0.0001) and the control (*F* = 49.43, *df* = 4, *p* < 0.0001).

### 3.4. Effect of Antigens on Pseudocapillary Formation

**Figure 9:**
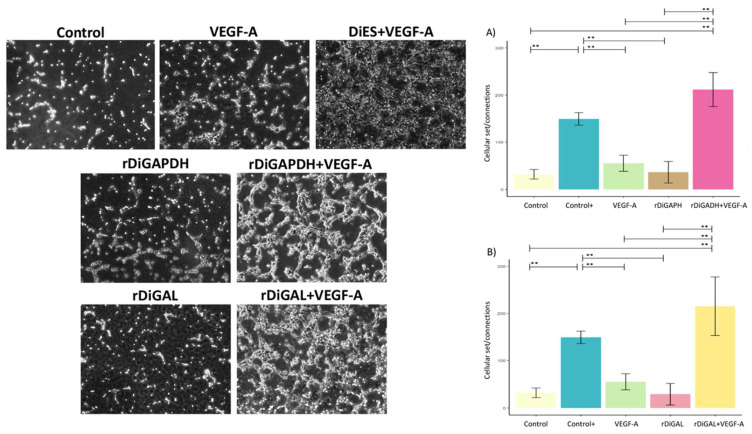
Representative images and effects of rDiGAPDH (**A**) and rDiGAL (**B**) on connections and cellular set in control (light yellow), Control+ (DiES+VEGF-A) (blue), VEGF-A (green), rDiGAPDH (brown), rDiGAPDH+VEGF-A (dark pink), rDiGAL (light pink), and rDiGAL+VEGF-A (dark yellow) during the first 90 min. Results are expressed as the means ± SDs of 3 independent experiments. Doble asterisk (**) indicate significant differences with *p* < 0.01.

Cultures treated with rDiGAPDH+VEGF-A showed significantly greater pseudocapillary formation and a higher cell junction-to-cell body ratio compared to those stimulated with rDiGAPDH (*F* = 40.1, *df* = 4, *p* < 0.0001) and VEGF-A (*F* = 40.1, *df* = 4, *p* < 0.0001) and the control (*F* = 40.1, *df* = 4, *p* < 0.0001) (Figure 9). No significant differences were observed between cultures stimulated with rDiGAPDH+VEGF-A and the positive control (*F* = 40.1, *df* = 4, *p* = 0.0564). A similar fact occurred with cultures treated with rDiGAL+VEGF-A. Those exhibited a significant increase in pseudocapillary formation compared to those stimulated with rDiGAL (*F* = 20.68, *df* = 4, *p* = 0.0002) and VEGF-A (*F* = 20.68, *df* = 4, *p* = 0.0007) and the control (*F* = 20.68, *df* = 4, *p* = 0.0002). However, no significant differences were found between cultures stimulated with rDiGAL+VEGF-A and the positive control (*F* = 20.68, *df* = 4, *p* = 0.1507). Significant differences were recorded between the positive control and cultures stimulated with VEGF-A (*F* = 40.1, *df* = 4, *p* = 0.0025), rDiGAPDH (*F* = 40.1, *df* = 4, *p* = 0.006), and rDiGAL (*F* = 20.68, *df* = 4, *p* = 0.006) and the control (*F* = 40.1, *df* = 4, *p* = 0.0004).

## 4. Discussion

Angiogenesis is a dynamic process in which new blood vessels are formed from existing ones. While it typically occurs during embryonic development, it can also be triggered in pathological conditions due to factors like hypoxia, inflammation, or tissue damage [1,41,42]. In cardiopulmonary dirofilariosis, adult *D. immitis* worms in the pulmonary artery produce proliferative endarteritis, the loss of elasticity, and pulmonary thromboembolism, leading to vessel inflammation and hypoxia [12,13,17,19,23,24]. When adult *D. immitis* worms die, proteins from the parasite and *Wolbachia* spp. are released into the bloodstream, leading to an exacerbation of the vascular endothelial inflammatory process [43,44]. However, through the production of excretory/secretory molecules, *D. immitis* is able to influence vascular endothelial physiology by stimulating vasodilation and limiting monocyte transmigration into vascular tissues, activating the host fibrinolytic system, and limiting clot formation, thus facilitating parasite survival [30,36].

Recently, it has been shown that *D. immitis* is also able to stimulate the proangiogenic pathway via excretory/secretory antigens (DiES) and associated surface molecules and cellular processes such as proliferation, migration, and pseudocapillary formation [38]. However, proteins of *D. immitis* somatic antigens and recombinant *Wolbachia* Surface Protein (rWSP) are able to promote the antiangiogenic pathway and do not stimulate, or even reduce, these cellular processes linked to the generation of new vessels [43,45].

Research on other filarial infections has provided evidence of lymphatic and blood vessel remodeling by cultured lymphatic endothelial cells exposed to live worms or *Wuchereria bancrofti* antigens. These antigens have induced the remodeling of lymphatic vessels, leading to cell proliferation and differentiation—processes linked to enhanced vascularization in damaged tissues [46]. Furthermore, several studies have indicated that angiogenesis and/or lymphangiogenesis may be stimulated by various factors released by adult monocytes activated by worms [47,48,49].

Our goal was to individualize the effects on the angiogenic process of two proteins present in DiES (rDiGAPDH and rDiGAL) that interact with the host fibrinolytic system. An in vitro model of endothelial cells was employed to replicate the onset of angiogenesis induced by the presence of adult *D. immitis* worms, which obstruct the pulmonary arteries and interact with the vascular endothelium. These recombinant proteins were produced while avoiding contamination with residual endotoxin traces. To verify this process, a test was performed to quantify their presence, yielding negative results in all cases. Since these residual traces can modulate the behavior of our model, a negative result suggests that the observed effects were not due to the presence of lipopolysaccharides in the analyzed assays.

VEGF-A is the main factor in the development of the angiogenic process and the first to occur [9]. For that reason, VEGF-A-stimulated endothelial cell cultures were used; these simulate the hypoxic condition and the initiation of angiogenesis, together with rDiGAPDH and rDiGAL proteins (rDiGAPDH+VEGF-A and rDiGAL+VEGF-A), and we also used cell cultures stimulated only with both DiES proteins (rDiGAPDH and rDiGAL). The results obtained showed that rDiGAPDH+VEGF-A and rDiGAL+VEGF-A promote the production of proangiogenic factors and the cellular processes of cell proliferation and migration and the formation of pseudocapillaries directly related to the proangiogenic pathway, thus participating in the survival of the parasite in the vascular endothelium.

On the one hand, we analyzed the effects of rDiGAPDH and rDiGAL on the production of VEGF-A (a key and initial factor in angiogenesis) and four molecules that initiate pro- (sVEGFR-2 and mEndoglin) and/or antiangiogenic pathways (VEGFR-1/sFlt1 and sEndoglin). Cell cultures treated with rDiGAPDH+VEGF-A and rDiGAL+VEGF-A exhibited a notable increase in the production of VEGF-A, sVEGFR-2 (a VEGF-A receptor that exerts a proangiogenic activity), and mEndoglin while no significant changes were observed in the production of VEGFR-1/sFlt1 and sEndoglin, which are known to have antiangiogenic effects. These results were observed in previous studies with respect to DiES and the *D. repens* somatic antigen [41,42]. In addition, endothelial cell cultures treated with the *D. immitis* somatic antigen and rWSP were also able to stimulate VEGF-A production under hypoxic conditions, but not to activate the proangiogenic pathway [43，^44^，^45^]. However, regarding the production of mEndoglin and sEndoglin, in previous studies, no alteration in the expression of any of the endoglin isoforms had been observed when endothelial cultures were treated with the *D. immitis* somatic antigen [50]. Thus, the results showed that in our in vitro endothelial cell model, stimulation with rDiGAPDH and rDiGAL supplemented with VEGF-A was able to stimulate the proangiogenic process, which could favor parasite survival in the host.

Finally, rDiGAPDH+VEGF-A and rDiGAL+VEGF-A produced a significant increase in cell proliferation and pseudocapillary formation and a significant decrease in cell migration (increased cell migration velocity) in our endothelial model, which was consistent with the induction of the proangiogenic pathway reported above. In an earlier study with DiES and *D. immitis* surface-molecule-associated antigens, Machado et al. [38] observed similar results. However, other studies have shown that, when the vascular endothelium is stimulated with the *D. immitis* somatic antigen, no changes in cellular processes occur [50], and even pseudocapillary formation is inhibited when endothelial cells are stimulated with rWSP [43,45]. This suggests that rDiGAPDH and rDiGAL, present in DiES, are involved in cellular processes that favor the survival of adult *D. immitis* worms until death, when *D. immitis* and *Wolbachia* spp. antigens are released in masse and the antiangiogenic pathway is stimulated.

## 5. Conclusions

This study showed the angiogenic capacity of rDiGAPDH and rDiGAL proteins, present in DiES, with VEGF-A in the vascular endothelium with in vitro endothelial cell cultures. Our results demonstrated the proangiogenic potential of rDiGAPDH and rDiGAL with VEGF-A by stimulating the production of proangiogenic factors and cellular processes related to angiogenesis. They also confirmed cellular processes directly associated with angiogenesis while interacting with the host fibrinolytic system, thus favoring the survival of *D. immitis* in the vascular endothelium. This study explored the complexity of the interaction between *D. immitis* and the vascular endothelium and the origin of how it may be able to survive for years in the host.

## Data Availability

The raw data supporting the conclusions of this article will be made available by the authors without undue reservation.
